# Lasing from a Large-Area 2D Material Enabled by a Dual-Resonance Metasurface

**DOI:** 10.1021/acsnano.4c00547

**Published:** 2024-05-06

**Authors:** Isabel Barth, Manuel Deckart, Donato Conteduca, Guilherme S. Arruda, Zeki Hayran, Sergej Pasko, Simonas Krotkus, Michael Heuken, Francesco Monticone, Thomas F. Krauss, Emiliano R. Martins, Yue Wang

**Affiliations:** † School of Physics, Engineering and Technology, University of York, York YO10 5DD, U.K.; ‡ São Carlos School of Engineering, Department of Electrical and Computer Engineering, University of São Paulo, São, Carlos-SP 13566-590, Brazil; § School of Electrical and Computer Engineering, Cornell University, Ithaca, New York 14853, United States; ∥ AIXTRON SE, Dornkaulstraße. 2, Herzogenrath 52134, Germany

**Keywords:** 2D material lasers, TMD, CVD, guided mode resonance, bound states in the continuum, stimulated emission, metasurface

## Abstract

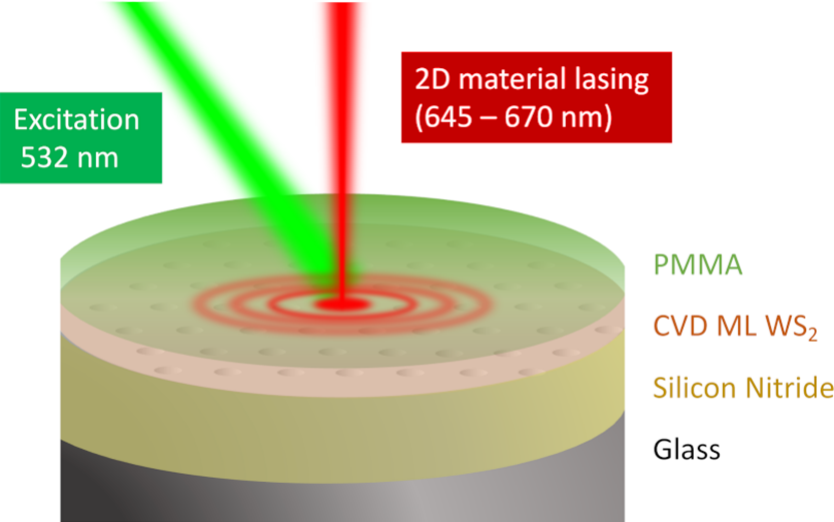

Semiconducting transition metal dichalcogenides (TMDs) have gained significant attention as a gain medium for nanolasers, owing to their unique ability to be easily placed and stacked on virtually any substrate. However, the atomically thin nature of the active material in existing TMD lasers and the limited size due to mechanical exfoliation presents a challenge, as their limited output power makes it difficult to distinguish between true laser operation and other “laser-like” phenomena. Here, we present room temperature lasing from a large-area tungsten disulfide (WS_2_) monolayer, grown by a wafer-scale chemical vapor deposition (CVD) technique. The monolayer is placed on a dual-resonance dielectric metasurface with a rectangular lattice designed to enhance both absorption and emission, resulting in an ultralow threshold operation (threshold well below 1 W/cm^2^). We provide a thorough study of the laser performance, paying special attention to directionality, output power, and spatial coherence. Notably, our lasers demonstrated a coherence length of over 30 μm, which is several times greater than what has been reported for 2D material lasers so far. Our realization of a single-mode laser from a CVD-grown monolayer presents exciting opportunities for integration and the development of real-world applications.

## Introduction

Since the first demonstration of room temperature vertical-cavity surface-emitting lasers (VCSELs) in 1988,^1^ tremendous progress has been made in the field of semiconductor nanolasers, driven by the desire to reduce the lasing threshold and develop more functionalities. Major breakthroughs are frequently linked to the discovery of modern materials that can amplify light and advancements in the fabrication processes of nanodevices. Recently, opportunities have arisen with the emergence of two-dimensional (2D) van der Waals (vdW) materials, especially the transition metal dichalcogenide (TMD) semiconductors^2,3^ and their heterostructures.^4,5^

Light sources based on 2D TMDs are particularly attractive due to their ability to be placed on a wide range of substrates, including wearables. The light emission properties of these materials are determined by excitons and trions, which result from strong Coulomb interactions.^6^ In particular, the excitons exhibit a strong binding energy of typically hundreds of meV, one or two orders of magnitude higher than, for example, gallium arsenide (GaAs) quantum wells, which leads to subnanosecond spontaneous emission lifetimes and stable room temperature operation. The underlying gain mechanism of 2D materials is complex. Recent work has revealed excitonic complexes and optical gain at densities 4–5 orders of magnitude lower than those of conventional semiconductors, which provides the foundation for low-threshold lasing devices.^7^ Furthermore, by stacking different monolayers of TMD vertically, the formation of vdW-bonded heterostructures is possible without encountering the typical “lattice mismatch” problem. This unique capability reveals extraordinary phenomena and enables the development of innovative optoelectronic devices, such as interlayer excitonic lasers that emit light at longer wavelengths into the infrared range.^8,9^

In order to realize these exciting opportunities, a number of issues need to be resolved: (a) limited absorption at the pump wavelength due to the nanometer-thin gain medium; (b) small mode overlap with the gain medium at the lasing wavelength for the same reason; and (c) the gain material’s lateral size is often limited, with mechanical exfoliation being the most common method for obtaining high-quality TMD materials until recently, which typically results in an active area limited to the micron scale. The exfoliated 2D TMDs are not suitable for large-scale production of devices for scalable technologies. Metal–organic chemical vapor deposition (MOCVD) serves as an alternative route toward growing large-area layered materials with high quality, low cost, and improved yield.^10^ High-performance diodes and transistors have been successfully demonstrated with wafer-scale TMDs,^11−13^ but lasers with CVD-grown TMDs have not yet been demonstrated. Here, we address these challenges by employing a design to resonantly enhance both the absorption at the pump wavelength and the emission at the lasing wavelength and by utilizing high-quality, large-area TMD monolayers grown by MOCVD.

Resonant enhancement has been used before, e.g., using high quality-factor (*Q*) whispering gallery modes,^14,15^ distributed-Bragg-reflector cavities,^16^ or photonic crystal cavities,^17^ but mostly at the emission wavelength only. Optical bound-states-in-the-continuum (BICs)^18^ have also been adopted for low-threshold lasing.^19−21^ Along similar lines, a “pseudo-BIC” tungsten disulfide (WS_2_) laser with a compact active region and external reflectors was recently demonstrated.^22^ It should be noted that all the resonator structures discussed above deliver extremely limited output power to the extent that absolute output power values are typically not reported. This low output also makes the characterization of laser performance extremely challenging, leading to a debate in the community as to whether lasing has been actually achieved.^23^ Although TMD resonant cavity emitters exhibit narrow line width, directionality, and polarization of emission, many studies have faced challenges in convincingly demonstrating the expected 2-fold line width reduction on threshold, spatial coherence, and output power, as previously mentioned. Additionally, the threshold behavior is typically subtle and can easily be mistaken for amplified spontaneous emission.

Here, we introduce a large-area TMD laser based on a dual-resonance metasurface for simultaneous absorption enhancement of the pump light and stimulation of the photoluminescence (PL) emission into a single lasing mode. The dual resonance addresses the limited absorption of 2D materials arising from their ultrathin nature, by deflecting photons horizontally and thus increasing light-matter interactions. By transferring a large-area CVD-grown monolayer onto such a metasurface, we demonstrate a monolayer laser with 10s nW output power. We characterize the lasing behavior and demonstrate exceptional temporal and spatial coherence that significantly exceeds the state-of-the-art in the field.

## Results

### CVD-Grown WS_2_ Monolayer

We choose WS_2_ as the active material not only because it has long-lived excitons^24,25^ and high gain^26^ compared to other TMD light-emitting materials but also because high-quality monolayer WS_2_ can now be grown on a wafer scale.^27,28^ The homogeneity of PL from an as-grown WS_2_ wafer is shown in Supporting Information 1.

### Dual-Resonance Design−the Rectangular Lattice

**Figure 1:**
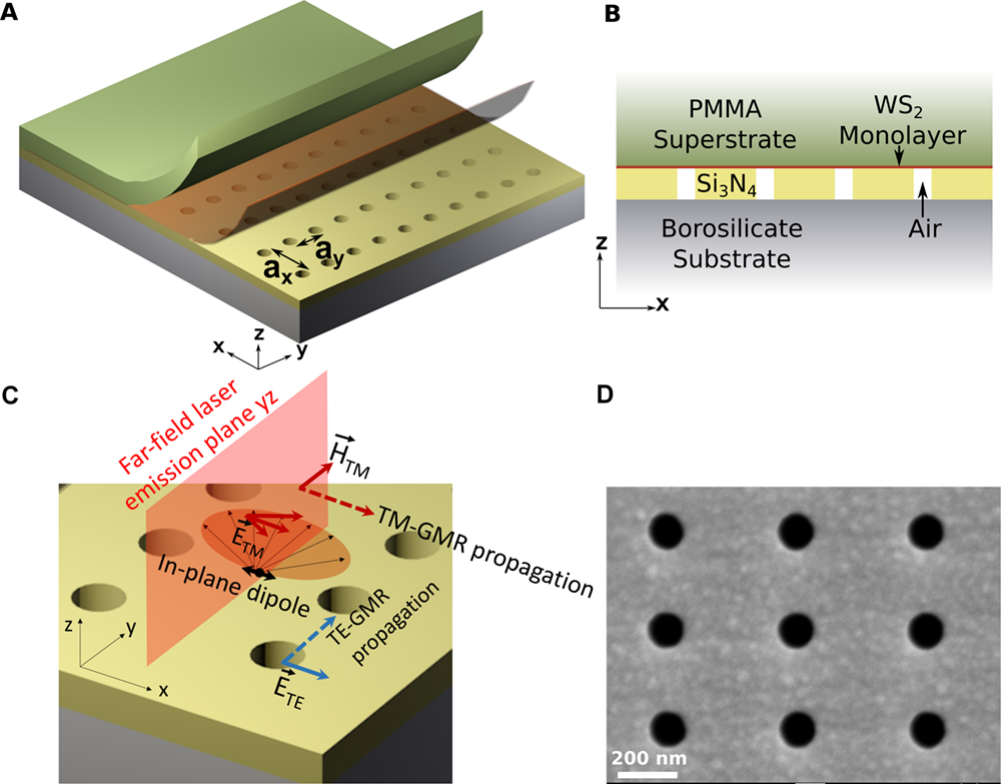
Dual-resonance nanohole array metasurface design and fabrication. (A) 3D rendering of the laser device, showing the rectangular lattice in Si_3_N_4_ with periods *a*_*x*_ and *a*_*y*_ > *a*_*x*_, the WS_2_ monolayer, and the PMMA layer. (B) Corresponding side view. (C) Schematic of the propagation and the field directions of the TE- and TM-like modes in the rectangular lattice. We excite the device with a TE-like mode, with the electric field (*E*_TE_) along the *x*-axis and the guided mode resonating in the *y* direction. The PL emission is coupled into a TM-like mode with the magnetic field (*H*_TM_) along the *y* direction and the TM-guided mode resonating in the *x* direction. The electric field of the TM mode is in the xz plane, which allows coupling with the in-plane dipoles in the WS_2_ layer. (D) SEM image of the fabricated bare Si_3_N_4_ metasurface (top view, *xy*-plane). Periods *a*_*x*_ = 410 nm and *a*_*y*_ = 320 nm, with hole diameter *d* = 140 nm.

**Figure 2:**
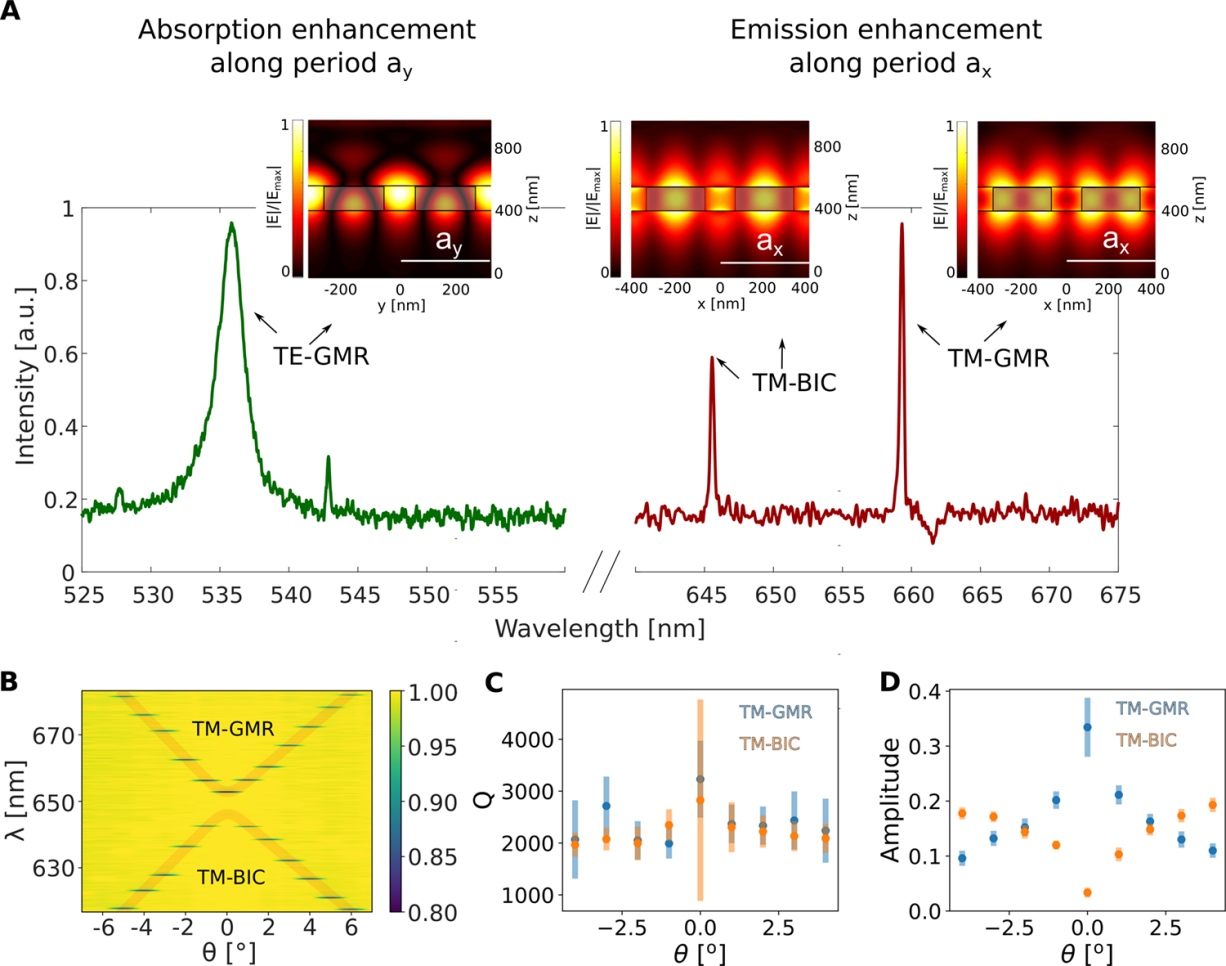
Experimental characterization of the dual-resonance metasurface. (A) Resonances supported by the rectangular lattice with the PMMA capping layer and without the WS_2_ layer, in the wavelength ranges of excitation (green) and emission (red). The peak around 535 nm (green curve) corresponds to a TE mode (dominant E-field component along *x*), while the peaks around 650 nm (red curve) correspond to the two TM modes, i.e., TM-GMR and TM-BIC with their field distributions plotted as insets. (B) Band structure of the two available modes for emission enhancement around normal incidence with an angular resolution of Δθ = 1°, measured in transmission. For the purpose of clearer visualization, both bands are plotted in faint red lines corresponding to the TM-GMR (top) and TM-BIC (bottom) modes, showing the vanishing of the symmetry-protected BIC mode around θ = 0° and a photonic band gap at around 650 nm. (C) *Q*-factor and (D) corresponding amplitude for both TM modes extracted from the transmission measurements with an angular resolution of Δθ = 1°. Note the error bar for the TM-BIC is very large at the Γ-point because of the weak signal.

To resonantly enhance both the absorption of the green pump laser (λ = 532 nm) and the PL emission of the WS_2_, we designed a dielectric nanohole array, in Si_3_N_4_-on-glass substrate (Figure 1), with the period in *y* (a_*y*_) suitably chosen to support a resonance for the pump wavelength and the period in *x* (*a*_*x*_) to support a resonance at the emission wavelength (see Supporting Information 2 for a detailed design and characterization of the metasurface). We deliberately chose a mode with a relatively low *Q* for the pump in order to increase the angular tolerance required for the excitation with a focused laser beam; specifically, we chose a transverse electric-guided mode resonance (TE-GMR), which results in a 2.6-fold enhancement in PL (simulation and experimental detail in Supporting Information 2.3). On the other hand, to achieve stimulated emission, we aim for the highest experimentally achievable *Q*-factor for a spatially extended resonant mode to enable narrow-bandwidth lasing and low-threshold operation. Two types of modes are available for this purpose, namely, the transverse magnetic-guided mode resonance (”TM-GMR”) and the symmetry-protected BIC TM mode (”TM-BIC”) (Figure 2), both of which provide a high *Q* of ∼3000 around the Γ-point (θ = 0°, see Supporting Information 3). The TM-BIC is a symmetry-protected mode with vanishing radiation loss at the Γ-point (more detail in Figure 2b and Supporting Information 2). We will later show that this symmetry-protected BIC mode and the TM-GMR mode can both support lasing around the Γ-point.

The feedback mechanism in our design is provided by band-edge resonances associated with the aforementioned guided modes of the photonic crystals.^29,30^ We note that although in an ideal structure (i.e., lossless and infinite size) the *Q*-factor at the Γ-point can be infinite in principle, however, in practical devices limited by scattering losses, the *Q*-factor of the TM-BIC mode becomes comparable to that of the TM-GMR mode (see Supporting Information 5).

The PMMA superstrate (Figure 1b) plays four important roles. First, it acts as a carrier to transfer the WS_2_ monolayer from its original growth substrate to the Si_3_N_4_ metasurface (see Methods). Second, it provides an advantageous mode overlap with the gain material because its refractive index (*n*_PMMA_ = 1.49) is higher than air, thereby drawing the mode up toward the superstrate (Supporting Information 4.2 and Figure S7). Third, the PMMA superstrate also results in a higher *Q*-factor. Fourth, it protects the active material during the fabrication process, slows down its degradation, and therefore prolongs the laser lifetime. In short, the PMMA layer is a critical component, as the absence of it results in the lack of any observed lasing activity.

### Laser Characterization: Threshold, Far-Field Radiation, and Spatial Coherence

**Figure 3:**
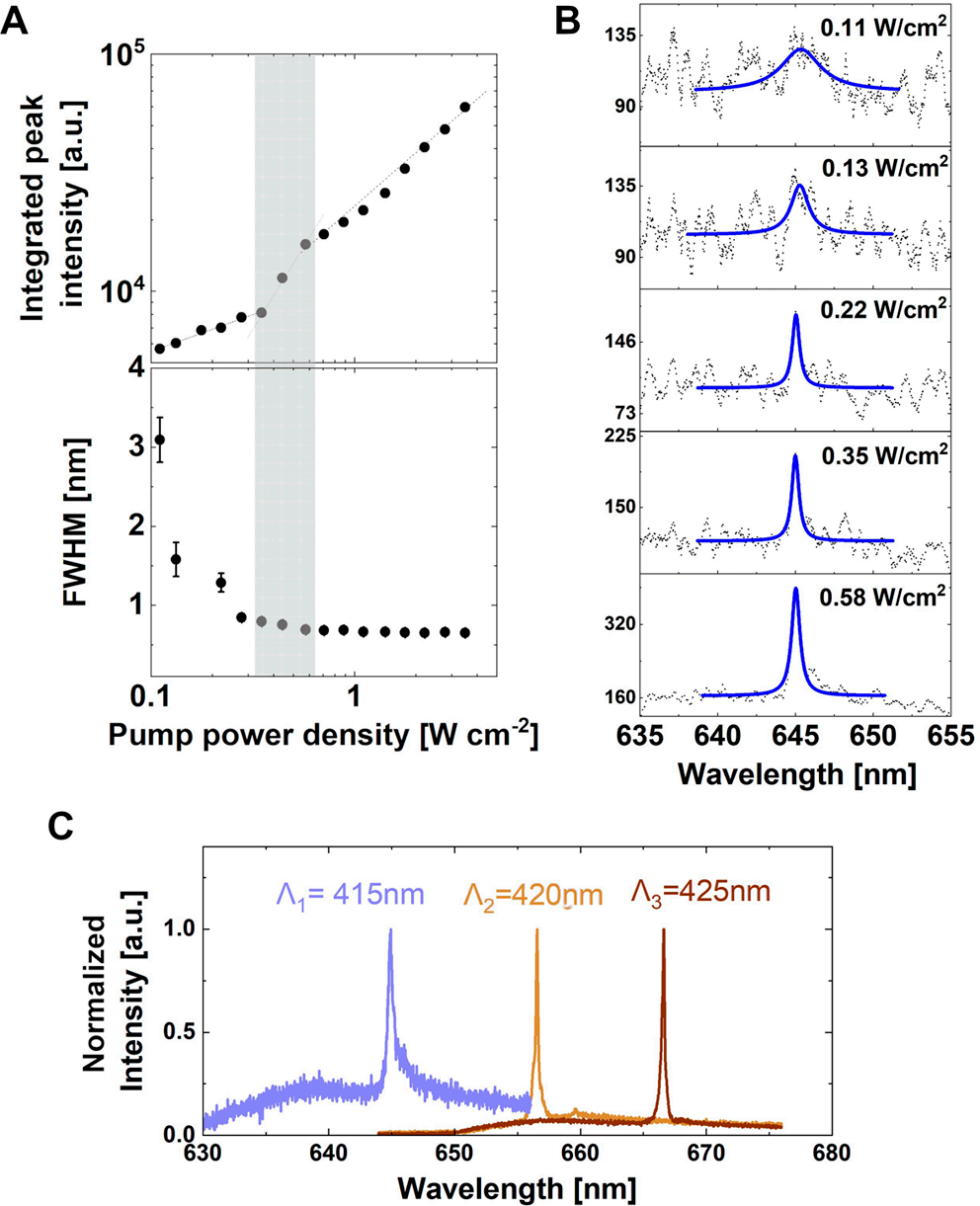
Laser threshold characterization. (A) Example of the threshold behavior (double logarithmic plot of the laser mode emission intensity with fullwidth at half maximum (FWHM) as a function of pump power density). Error bars in the line widths are extracted from the Lorentzian fits. In the grey area, there is a combination of stimulated and amplified spontaneous emission. Above 0.58 W/cm^2^ pump power density (intensities above the shaded area), the lasing is fully established and the FWHM of the laser peak is 0.6 nm. (B) Emission spectra (black dotted lines) with different pump power densities below and near the threshold, as well as the fitted curves (solid blue lines) to extract the FWHM of the peak. (C) Tuning of the lasing wavelength by changing the long period, *a*_*x*_ = 415 (blue), 420 (orange), and 425 nm (red). Their L–L curves and further lithographic tuning analysis are included in Supporting Information 9.

We used lithographic tuning to establish the best combination of *a*_*x*_ and *a*_*y*_ values and constructed a matrix covering the range of *a*_*x*_ = 400–425 nm and *a*_*y*_ = 320–325 nm to match both the pump laser and the WS_2_ emission wavelength. Out of the 15 devices characterized, we observed laser emission typically occurring at wavelengths above 645 nm, where the extinction coefficient of the WS_2_ is sufficiently low to avoid the reabsorption of emitted photons (see Supporting Information 9), in accordance with previous reports.^14^ Low threshold densities and significant linewidth reductions were observed in all samples; see Figure 3 as an example. We typically observed a linewidth reduction by a factor of 2–3, in line with the established laser theory.^31^ At low pump power, the signal-to-noise ratio is small, which introduces fitting uncertainty in that range. On the other hand, at high pump power levels, we measured an output power in excess of 20 nW. The measured output power is naturally lower than the total emitted power due to factors such as the collection lens with a numerical aperture (NA) of 0.1, which cannot capture all the light, and the presence of a 50/50 beam splitter in the collection path. As a result, we estimate that the actual laser power is at least 100 nW (see Methods). Further discussion on estimated output power fulfilling the quantum threshold condition is included in the Supporting Information 6.

**Figure 4:**
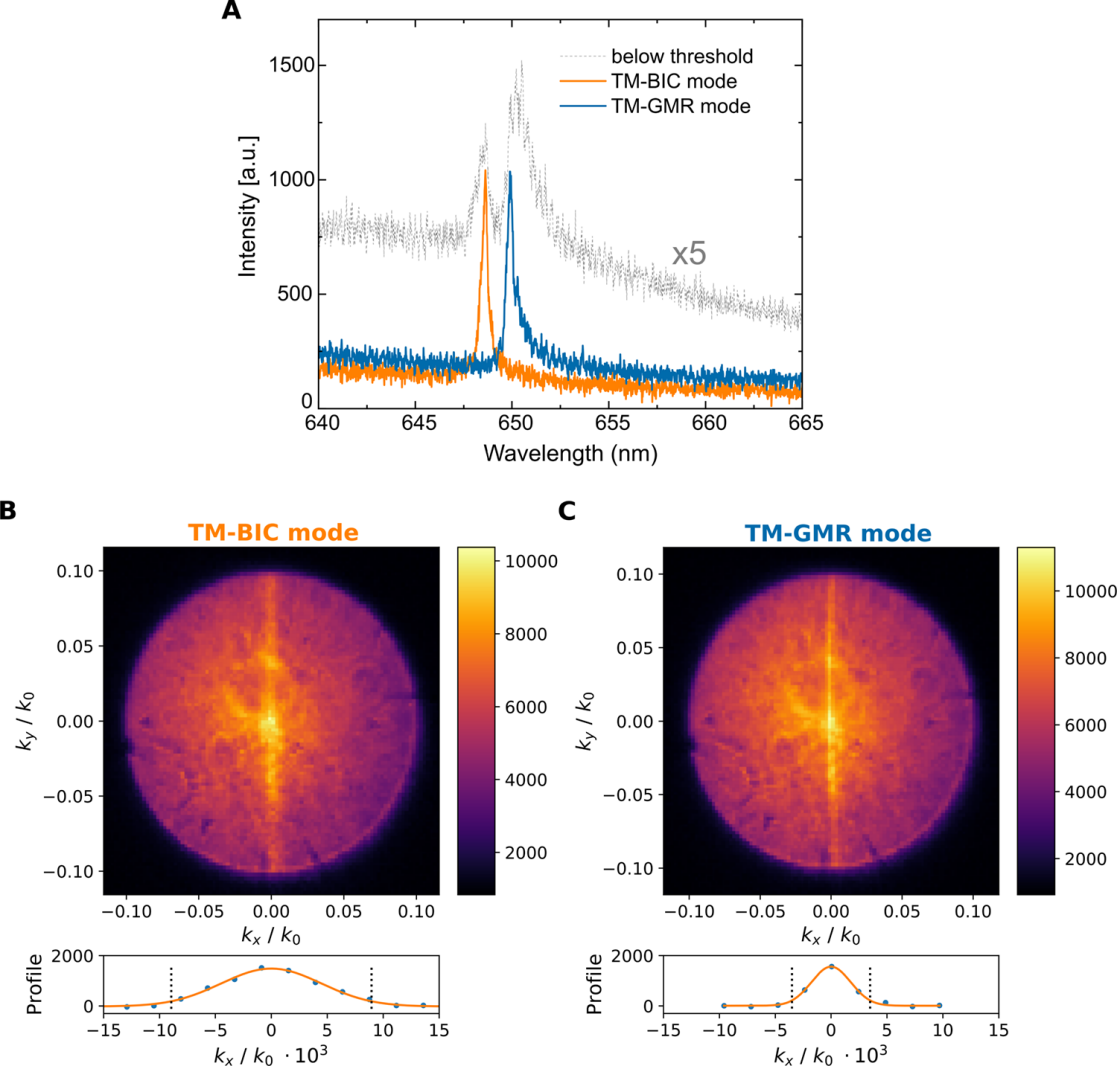
Lasing modes and their far-field patterns. (A) Emission spectra below (×5, black dotted line) and above threshold, with lasing at the TM-BIC (orange line) and the TM-GMR (blue line) modes. The pump power densities are 0.28 and 1.45 W/cm^2^, respectively. (B, C) (top) Far-field emission patterns for the TM-BIC and TM-GMR modes, respectively. (bottom) An average profile along *k*_*x*_ is obtained and baseline-corrected, as described in Figure S13. Black dotted lines are plotted at *k*_*x*_ = ± 2σ, with σ obtained from a Gaussian fit. More information can be found in Supporting Information 8.1.

The utilization of both the rectangular lattice and the high refractive-index superstrate discussed above is critical for achieving lasing at low pump power and for producing significant output power. Indeed, lasing was only observed with doubly resonant metasurfaces (i.e., no lasing was observed when the metasurface was not resonant at the pump wavelength). Another consequence of using a rectangular lattice instead of a square lattice is that it produces a line-shaped far-field emission pattern (Figure 4b,c). The observed shape is typical of lasers that exhibit feedback in a single direction. This is precisely the case with our rectangular lattice. The resonant emission enhancement is attained with a one-dimensional periodicity, resulting in a narrow laser beam along the *x*-direction of the lattice. In contrast, in the *y* direction, the lasing mode can couple with a broad range of angles. Figure 4 shows lasing with the TM-GMR mode and the TM-BIC mode in the same device, along with their far-field emission patterns, respectively. Along the *x* direction, the divergence of the TM-BIC laser (2σ = 8.8 mrad) is approximately 2.5 times the divergence of the TM-GMR laser (2σ = 3.5 mrad). The larger divergence of the TM-BIC laser along the *x*-axis, compared to the TM-GMR laser, can be attributed to the inhibited radiation at the Γ-point. For both modes, the divergence angle along the *y* direction is significantly larger than the numerical aperture of our objective (NA = 0.1), which means that the measured output power value substantially underestimates the total emission power. The corresponding spectra below and above the threshold are shown in Figure 4a.

**Figure 5:**
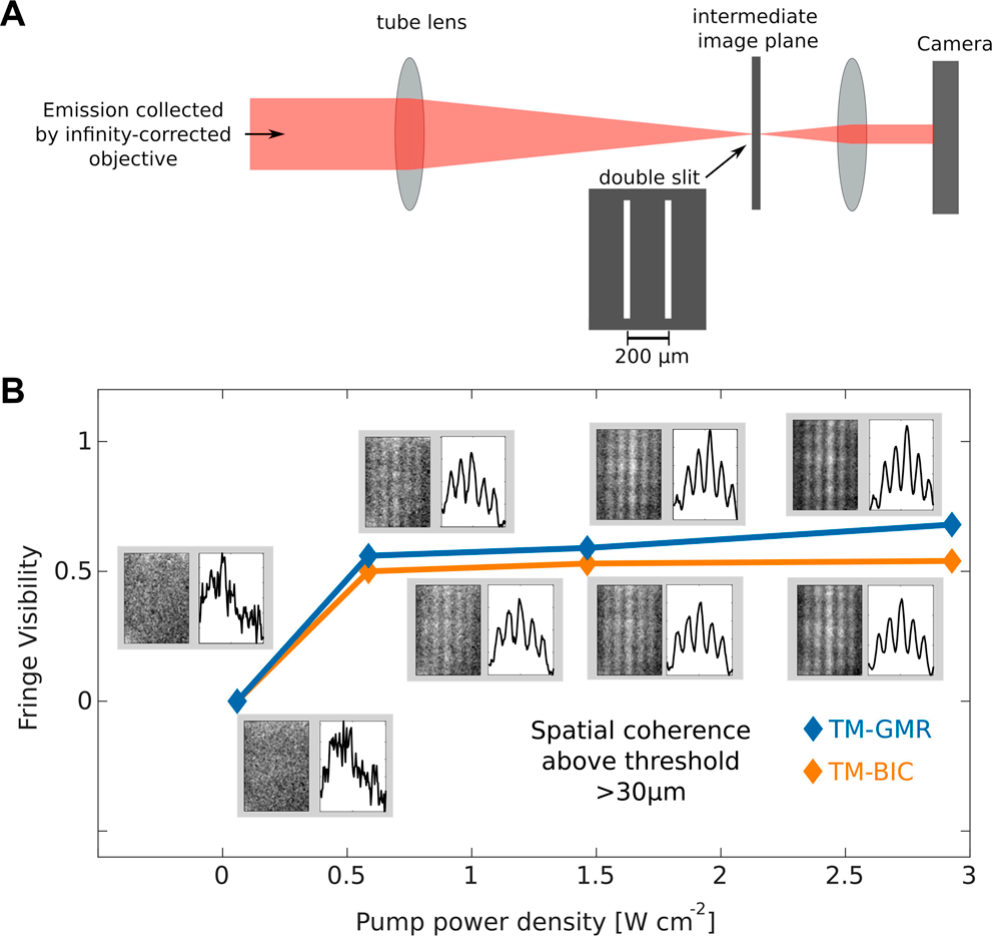
Characterization of spatial coherence. (A) Schematic of the double-slit placed into an intermediate image plane (in addition to the micro-PL setup, see Supporting Information 2.2) and (B) interferograms recorded below and above threshold with a double slit distance of 200 μm, corresponding to a distance of 30 μm on the sample. The interferograms were recorded with the same integration time at four different pump power values for both types of laser.

To characterize the spatial coherence of our lasers, we use Young’s slit method^32^ by placing a double-slit in the intermediate image plane and projecting the resulting interference pattern onto the camera (Figure 5). Above the threshold, the interferograms show clear fringes with a visibility of at least 0.5 (see Figure 5b and Methods) for a double slit spacing of 200 μng the magnification of the intermediate image plane into account, this spacing corresponds to a distance of 30 μm in the sample plane. We, therefore, conclude that the light emitted by the 2D material laser, for both the TM-GMR and the TM-BIC modes, is spatially coherent over distances of at least 30 μm, which is higher than reported for any comparable device.^9,33,34^ Another double-slit spacing of 300 μm is also used to measure the coherence below and above the threshold (see Supporting Information 8.2). Although we see a significant difference in the interferograms measured below and above the threshold, the visibility with the 300 μm slit is worse than the 200 μm slit.

## Conclusions

We have presented comprehensive evidence of lasing from a 2D material, enabled by a dielectric metasurface with a doubly-resonant design. The rectangular array allows for the enhancement at both the pump and the emission wavelength of the laser, resulting in room-temperature lasing with a noteworthy low threshold of below 1 W/cm^2^. Specifically, the lattice constant in one direction was chosen to resonate with the pump, while the lattice constant in the other direction supports high *Q*-factor (*Q* ∼ 3000) TM-polarized resonances to enhance laser emission. Notably, the two types of lasing modes we observed, i.e., the TM-GMR mode and the TM symmetry-protected BIC mode, exhibit similar threshold values and far-field emission patterns.

The resonant enhancement of both the pump and the PL emission, together with the large active area of the laser, enabled by the wafer-scale MOCVD-grown WS_2_ monolayer, allows us to obtain a very low-threshold pump power density and an output power of tens of nW without damaging the active material (pumped at 20× the laser threshold). This relatively high output power was essential for a complete characterization of the 2D material-based lasing devices, especially concerning the far-field characterization, the spatial coherence, and the quantum threshold condition. Notably, the threshold and output power we observe compare very favorably to other TMD devices (see Table 1, where we present a selection of best performing devices reported in the literature. All characteristics were obtained at room temperature, except for ref (13), where lasing was achieved at 130 K maximum). We emphasize that the interplay between the coupling-in of the excitation and the coupling-out of the emitted light with different modes together determines the laser performance. Investigation of the optimal pump spot can be an interesting follow-up work.

**Table 1:** Laser Performance Comparison

**resonance category**	**active material (“/” with capping)**	**threshold** (W/cm^2^)	**pump area** (μm^2^)	**extracharacterization**^a^
defect-type cavity^17^	1L WSe_2_	1	2.69	reproducibility
defect-type cavity^8^	1L MoS_2_-1L WSe_2_	2548	0.785	first-order coherence
guided mode resonance^9^	1L WSe_2_–1L MoSe_2_	22	approximately 0.8	far-field emission; first-order coherence
VECSL^16^	1L WS_2_	0.442	1.13	
nanobeam^42^	hBN/1L MoTe_2_/hBN	6.6	4.67	
BIC^22^	1L WS_2_/CYTOP	144	0.95	far-field emission
microsphere (MP)^43^	2L WSe_2_/MP	0.72	1.56	
microdisk^15^	4L MoS_2_	630	0.785	
whispering gallery modes^39^	50 nm WS_2_ disk	1250	38.5	far-field emission; second-order coherence
our work	MOCVD 1L WS_2_/PMMA	0.2–0.5	18,626.5	far-field emission and output power; first-order coherence; tunability

a Laser characterization beyond the L-L threshold behavior and linewidth narrowing.

Our lasers demonstrated a coherence length of over 30 μm, which is several times greater than what has been reported for 2D material lasers. The upper bound of the spatial coherence length estimated from the angular spread presented in the far-field images, see Supporting Information 8.3, was even higher than the measured coherence. Coherence is not only an important fundamental property of a laser source but also a desirable feature in many technologies. For example, the highly coherent and polarized states from these devices can be useful for improving stability and security in quantum communication.^35,36^ Coherence can also enable next-generation label-free biosensing with on-chip interferometry, which has the potential for ultrahigh sensitivity and robustness.^37,38^ We note here that the coherence we measured here and in some of the works in Table 1 are all first-order coherence. Second-order correlation function, *g*(2), measurement could add further value to the laser characterization.^39^

Compared to optically pumped devices, 2D material light emission via electrical injection is more challenging. Recent work revealed another gain mechanism involving charged excitons or trions in electrically gated monolayer and bilayer MoTe_2_, at densities 4–5 orders of magnitude lower than the Mott density.^7^ The closest attempt toward electrically pumped lasing in 2D material is by embedding a WSe_2_ electrically injected LED inside a microcavity^40^; however, only directionality and brightness improvement have been demonstrated, but not laser operation. With ongoing efforts in improving light-matter interaction and optimizing electrical contacts, we believe that electrically injected 2D material lasers are on the horizon.

Our approach of absorption enhancement combined with emission enhancement in a rectangular lattice nanohole array, together with the large active area enabled by the extended guided-mode resonances and the MOCVD-grown material, is readily applicable to other light-emitting TMDs and their heterostructures. Hence, the lasing operation can be readily extended to other wavelength ranges. Such dual resonances can also be utilized with other types of gain materials, including organic semiconductor thin films.^41^ In conclusion, with our successful demonstration of practical emission powers and the potential for wafer-scale production, we anticipate that our research will lead to the implementation of 2D semiconductor light sources on heterogeneous substrates, such as flexible substrates/wearables. Additionally, our work holds promise for the advancement of biological and chemical sensing, as well as quantum optics and communication technology platforms.

## Methods

### Design Simulations

For the design of the nanohole array,^44^ especially for identifying suitable periods to support the desired resonances, we simulated the resonances and their field components using the Rigorous Coupled Wave Analysis (RCWA) method.^45^ For the refractive indices of the glass substrate, the metasurface material (Si_3_N_4_), and the polymethyl methacrylate (PMMA) polymer, we used *n*_glass_ = 1.46,^46^*n*_Si_3_N_4__ = 2.04,^47^ and *n*_PMMA_ = 1.49,^48^ respectively. We assumed the values to be constant within the wavelength range of interest. We also carried out 3D Finite Element Method simulations (COMSOL Multiphysics) to verify the performance of the nanohole array metasurfaces. The results obtained from these simulations were consistent with the RCWA simulations (see Supporting Information 4).

### Metasurface Fabrication

We purchased glass wafers coated with silicon nitride (150 nm Si_3_N_4_ on 500 μm glass). These wafers were patterned by electron beam lithography (EBL) with a resist layer (AR-P 6200.13, Allresist GmbH) and a charge dissipation layer (AR-PC 5090, Allresist GmbH). We use an EBL system with an acceleration voltage of 50 kV (Voyager, Raith GmbH) to define the nanohole array pattern. We etch the pattern into the Si_3_N_4_ layer employing reactive ion etching, using a mixed gas flow of CHF_3_ and O_2_ (29:1). In a final step, we remove the resist residue in 1165, a resist remover, leaving the metasurface ready for transferring the TMD gain material.

### Band Diagram of Cold Cavities

To characterize the cold cavity modes of the rectangular nanohole array before the WS_2_ monolayer is applied, we spin-coat the sample with a 400 nm thick PMMA layer. The band structure is measured in transmission on a rotation stage with a collimated visible light source and a high-resolution spectrometer (Acton Spectrapro 2750). The transmission spectra are acquired by tilting the sample across a range of angles with respect to the beam. The resulting spectra are normalized by the thin film response of the bare Si_3_N_4_ substrate.

### WS_2_ Laser Fabrication

Monolayer WS_2_ is grown on a 2-in. sapphire substrate by AIXTRON Ltd. and AIXTRON SE using a Close Coupled Showerhead metal–organic vapor deposition reactor. The uniformity of as-grown monolayer WS_2_ across the 2-in. wafer is assessed using photoluminescence and Raman spectroscopy, as well as AFM and SEM. Centimeter-sized monolayers of WS_2_ are lifted from the sapphire substrate with buffered oxide etching (8 min) and then transferred from the sapphire substrate onto pre-patterned metasurfaces, using a wet-transfer process with a 400 nm thick PMMA layer as a carrier. The PMMA layer consequently acts as a superstrate and an encapsulation layer in the laser devices. The size of a single device is typically 500 × 500 μm, and we usually fabricate 25 devices with different designs on each substrate (1 × 1 cm).

### Photoluminescence and Power Measurements

We use a micro-PL setup (Supporting Information 2) to excite the devices with a CW laser (Novanta Photonics gem532) via a 4× objective (NA = 0.1) and collect the emission via the same objective. For the threshold characterization, we record spectra with a range of pump powers. The excitation power on the sample is measured with a Thorlabs power meter (photodiode sensor model S121C). Various pump powers are obtained by inserting different optical neutral density (ND) filters in the excitation path. The excitation intensity is calculated with the reference power and the density of the ND filters. The emission power is measured after the 50/50 beamsplitter, with a silicon low-power laser probe (Gentec-eo Pronto-Si, noise level 10 pW, the measured power is calibrated at 633 nm). The actual power from the laser device is estimated to be at least five times the measured power, taking into account the beam splitter, the objective NA, and the large beam divergence along the *y* direction (the “fan” shape of the emitted beam).

### Spot Size of the Excitation Laser Beam

In order to determine the spot size of the excitation laser spot on the sample, we focus a real space image of the laser spot on the camera (CoolSNAP Myo) with a lens placed at its focal distance in front of the camera. The imaged spot is fitted with a 2D-Gaussian distribution, and the spot radius (radius = 77 μm) is taken as *r* = 2σ, corresponding to the radius at which the intensity drops by 1/*e*^2^ of its peak value. We calculate the pump beam to be 18 626.5 μm^2^ in area (see Table 1). We note that the pump spot is deflected and coupled into the sample plane with the TE-guided mode, which means that the effective pump area is larger than the pump beam.

### Measurement of the Far-Field Radiation Pattern

The far-field radiation patterns were measured by imaging the back focal plane (BFP) of the objective onto the camera. To ensure that only the PL emission is captured and the excitation light is blocked, we utilize a Thorlabs FEL0550 long-pass filter. The axes of the resulting image of the BFP focused on the camera are proportional to the in-plane wave vector components *k*_*i*_ = *k*_0_ sin(θ_*i*_), for *i* ∈ *x*, *y*. The pixel number along each axis is converted to *k*_*i*_ by centering and multiplying with an appropriate calibration factor, which is found via the radius of the NA-circle.

## Data Availability

The authors declare that all the data and code supporting the findings of this study are available within the article or upon request from the corresponding author. The main results in this study are available in the Research Square preprint repository.^49^

## Supporting Information Available

Uniformity of CVD-grown monolayer WS_2_, transmission band diagram simulation and measurement, lasing on the Γ point vs lasing off the Γ point, PMMA effect on field confinement and *Q*-factor, *Q*-factor effected by finite size and loss, output power and quantum threshold condition, laser polarization in the far field, coherence and far-field analysis, and wavelength tunability (PDF)
