# Supplementary material for: Lasing from a Large-Area 2D Material Enabled by a Dual-Resonance Metasurface
Source: ACS Nano. 2024 May 6;18(20):12897–904. doi: 10.1021/acsnano.4c00547 (PMC11112975; doi:10.1021/acsnano.4c00547)
Supplement: Supplementary file 1 — nn4c00547_si_001.pdf [file nn4c00547_si_001.pdf]

Lasing from a large area 2D material enabled by a dual-resonance metasurface

Isabel Barth[1,+], Manuel Deckart[1,+], Donato Conteduca[1], Guilherme S Arruda[2], Zeki Hayran[3], Sergej Pasko[4], Simonas Krotkus[4], Michael Heuken[4], Francesco Monticone[3], Thomas F Krauss[1], Emiliano R Martins[2], and Yue Wang[1,*]

[1]Department of Physics, School of Physics, Engineering and Technology, University of York, York, YO10 5DD, United Kingdom
[2]São Carlos School of Engineering, Department of Electrical and Computer Engineering, University of São Paulo, São Carlos-SP 13566-590, Brazil
[3]School of Electrical and Computer Engineering, Cornell University, Ithaca, New York,14853, USA
[4]AIXTRON SE, Dornkaulstraße. 2, 52134 Herzogenrath, Germany
[*]corresponding author email: yue.wang@york.ac.uk
[+]these authors contributed equally to this work

ABSTRACT

Semiconducting transition metal dichalcogenides (TMDs) have gained significant attention as a gain medium for nanolasers, owing to their unique ability to be easily placed and stacked on virtually any substrate. However, the atomically thin nature of the active material in existing TMD lasers and the limited size due to mechanical exfoliation presents a challenge, as their limited output power makes it difficult to distinguish between true laser operation and other "laser-like" phenomena. Here, we present room temperature lasing from a large-area tungsten disulphide (WS_2) monolayer, grown by a wafer-scale chemical vapor deposition (CVD) technique. The monolayer is placed on a dual-resonance dielectric metasurface with a rectangular lattice designed to enhance both absorption and emission; resulting in an ultralow threshold operation (threshold well below 1 W/cm^2). We provide a thorough study of the laser performance, paying special attention to directionality, output power, and spatial coherence. Notably, our lasers demonstrated a coherence length of over 30 μm, which is several times greater than what has been reported for 2D material lasers so far. Our realisation of a single-mode laser from a CVD-grown monolayer presents exciting opportunities for integration and the development of real-world applications.

Supplementary Information

1 Uniformity of CVD grown monolayer WS_2

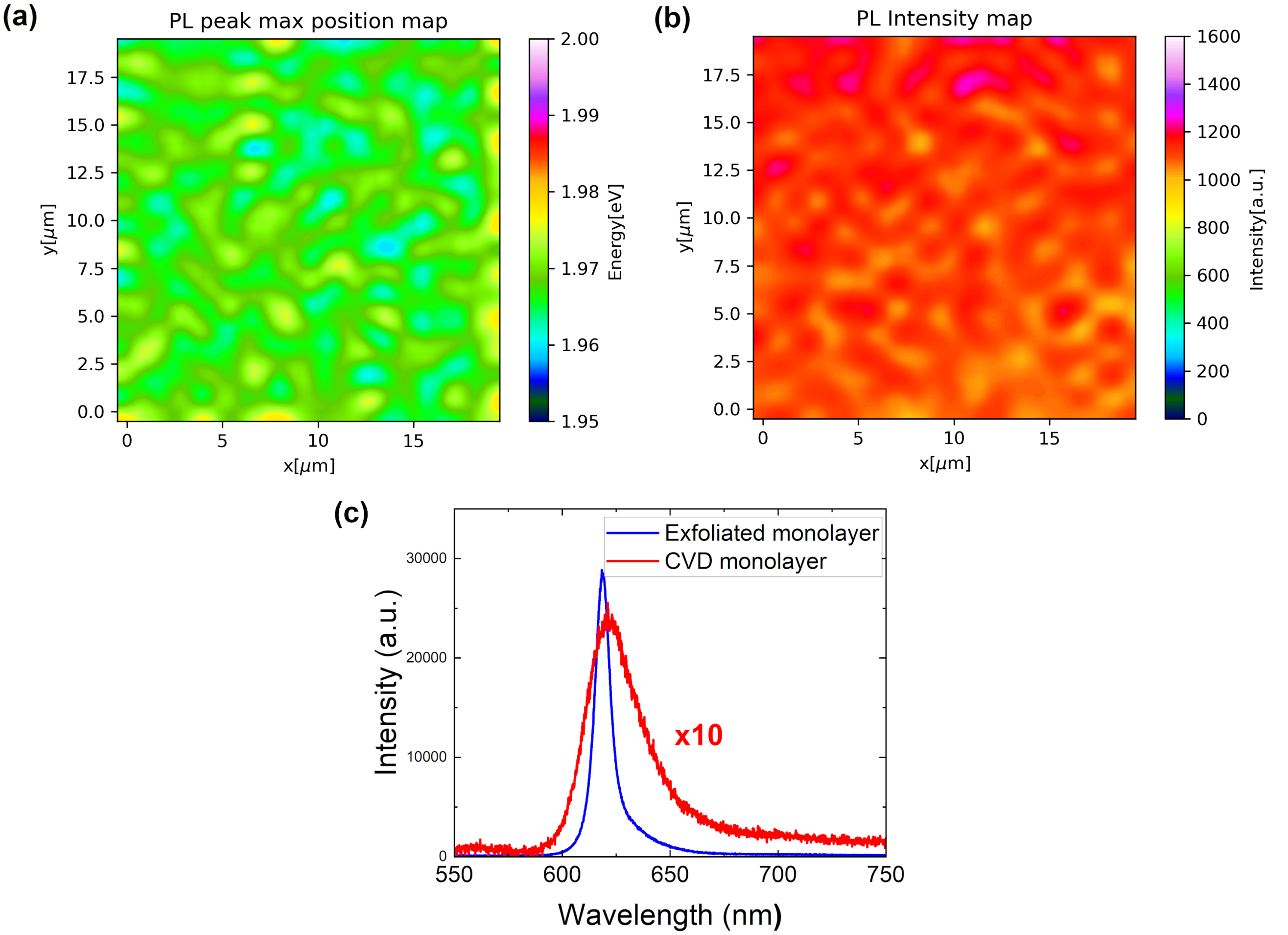

Figure S1. PL of a CVD-grown monolayer on a 2 inch sapphire wafer, compared to an exfoliated monolayer flake <10 μm. a) PL peak position mapping over a 20x20 μm area of the as-grown monolayer; **b)** Uniformity of PL intensity over the same area; **c)** PL spectra of the CVD-grown monolayer (red curve) and the exfoliated monolayer (blue curve), excited with a 1.5 μm diameter pump spot. The integrated PL intensity of the exfoliated monolayer is approximately 4 times higher than the CVD-grown monolayer.

2 Transmission Band Diagram simulation and measurement

2.1 Transmission Band Diagram simulation

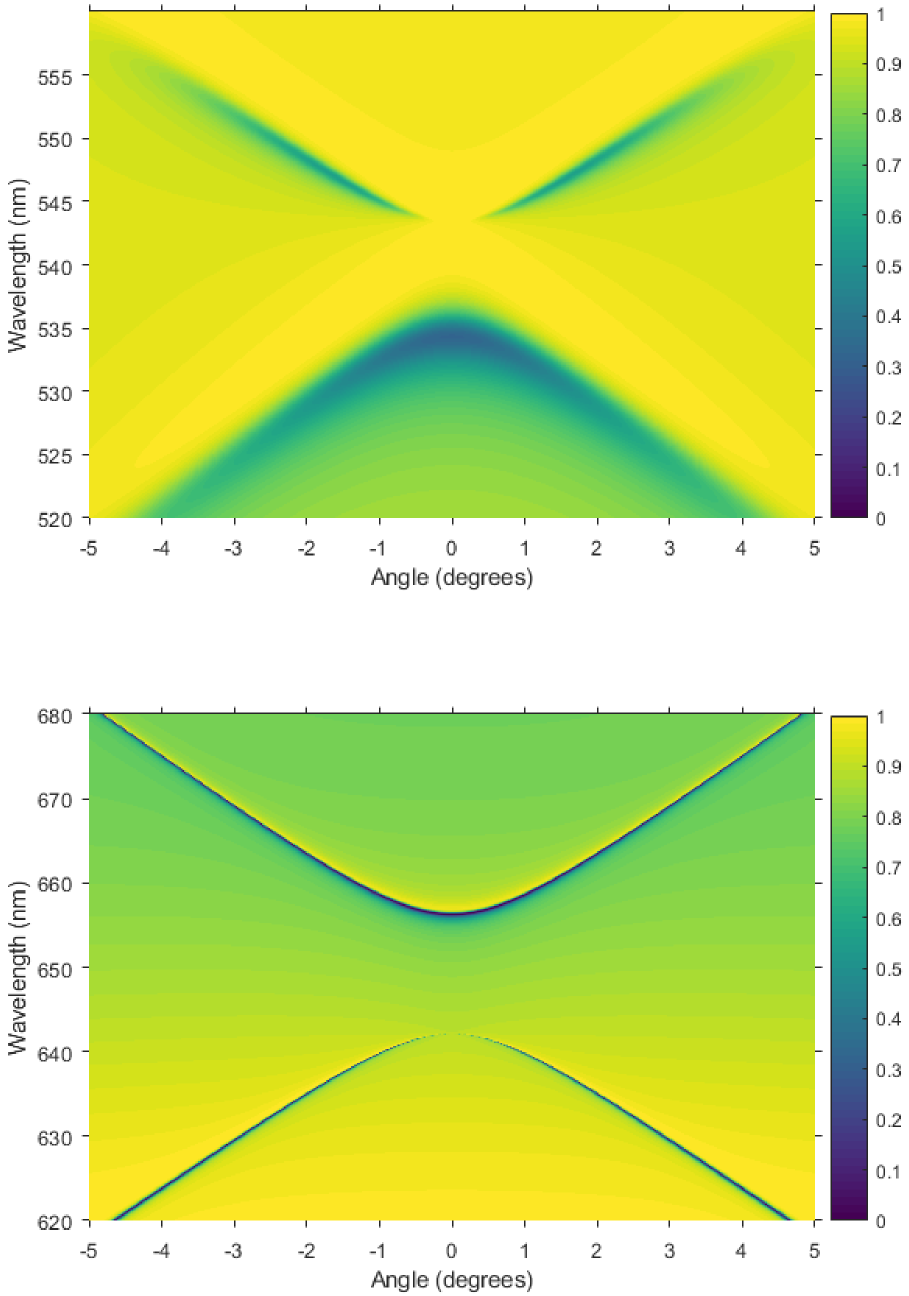

Figure S2. Transmission band diagram simulation: top) around the excitation wavelength; **bottom)** around the emission wavelength.

2.2 Experimental setup

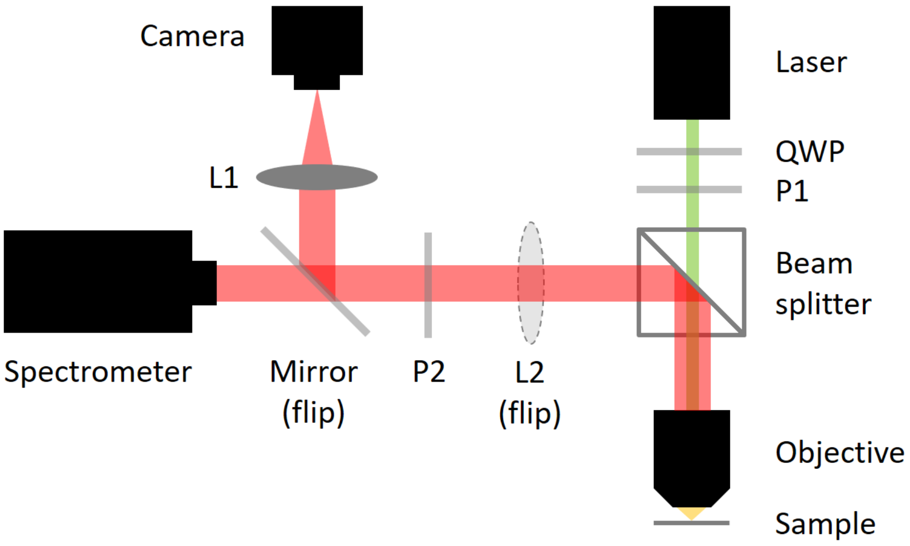

Figure S3. Mirco-PL setup schematic. A collimated laser beam ($\lambda = 532$ nm) is sent through a beam splitter into the objective to form a loosely focused spot on the sample. The PL from the sample is collected by the objective, reflects at the beam splitter and is analysed by a spectrometer (Acton Spectrapro 2750). A mirror on a flip mount can be inserted into the beam path to direct the light through a camera lens (L1), which forms an image on the camera sensor (CoolSNAP Myo). A second lens (L2) can be flipped into the beam path in order to image the back focal plane of the objective.

2.3 PL Enhancement (below threshold)

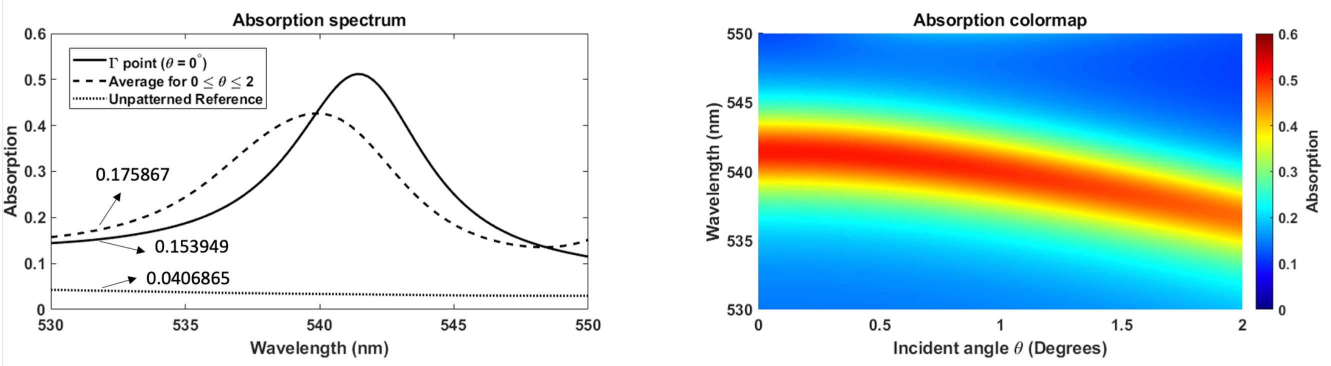

Figure S4. Resonance enhancement at the pump wavelength. **a)** RCWA simulation of the resonance enhancement around the pump wavelength 532 nm, showing approximately 4 folds enhancement with the metasurface compared to non-structured surface. **b)** TE-GMR resonance at a range of incidence angles.

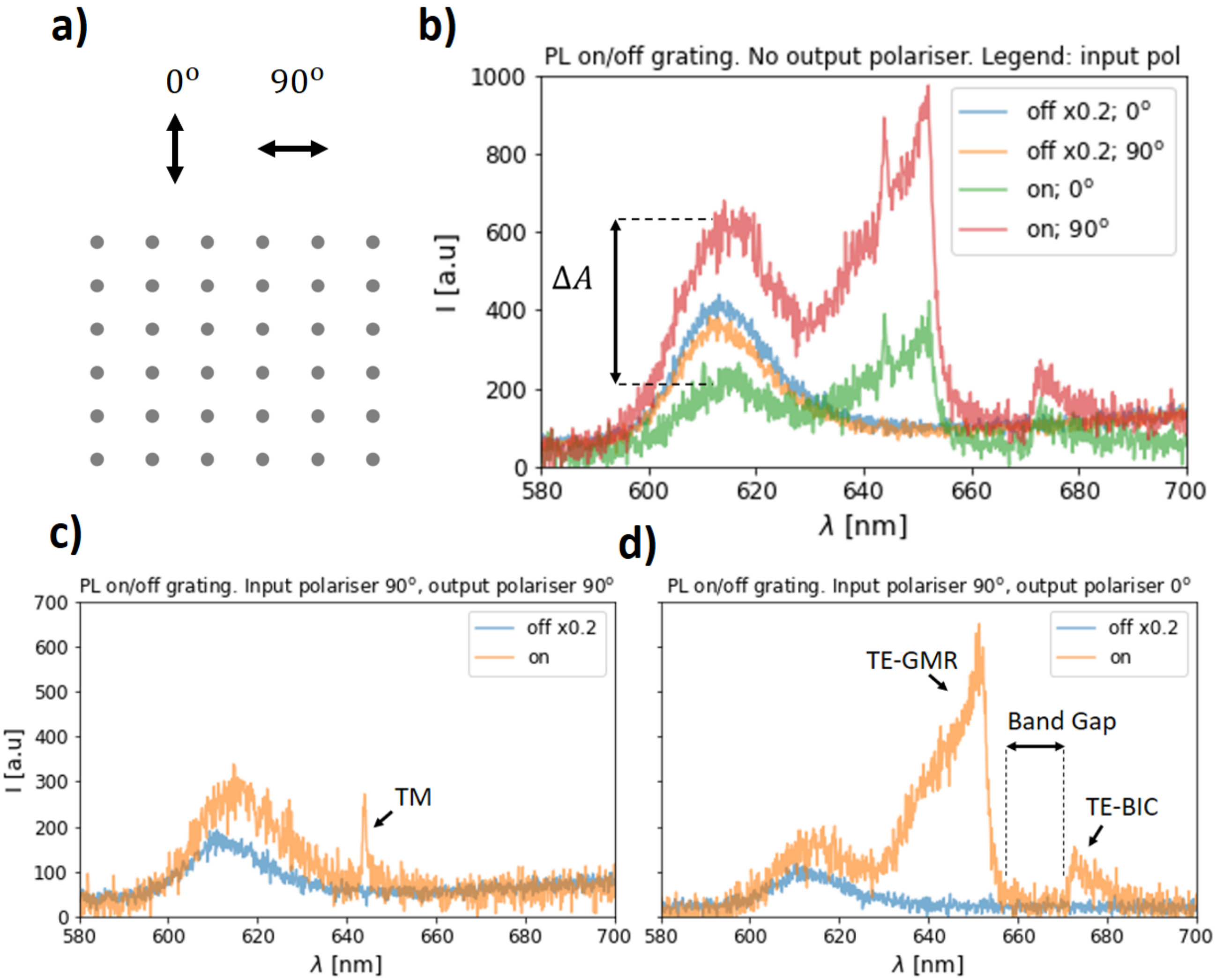

Figure S5. PL enhancement below lasing threshold with rectangular lattice (periods $a_x = 405$ nm and $a_y = 325$ nm). **a)** Orientation of the polarisers with respect to the rectangular nanohole array. **b)** Spectra of PL on/off grating with polariser on the excitation side, without any polariser on the collection side. The integrated spectrum in the range $\lambda \in [590, 690]$ nm is ~ 2.6 times higher for the resonant polarisation (90°). Off-grating spectra have been integrated for 10 s and are multiplied by a factor of 0.2, in order to allow comparison with the on-grating spectra, for which the integration time is 2 s. **c)** Spectrum with polariser on the collection side at 90°, revealing the TM mode at 642 nm. **d)** Collection polariser at 0°, revealing both the TE-GMR and the TE-BIC mode, separated by the photonic band gap.

3 Lasing on the Γ point vs lasing off the Γ point

Typically, DFB lasers are favoured to lase on the Γ point. With the advent of BIC, however, there has been a surge in interest in taking advantage of high Q resonances off the Γ point, the so-called accidental BICs[1]. Here we briefly discuss the advantages of the Γ point for lasing.

For the sake of simplicity, we assume a one directional periodical structure. At the Γ point, the dominant components of the Bloch mode B_0 are:

$$B_0 = A_{-1}e^{-iGx} + A_0 + A_1 e^{+iGx}$$

The components A_{-1} and A_1 represent counter-propagating waves which are coupled by the structure's periodicity. These are the lasing components.

We now consider two symmetric Bloch modes off the Γ point: :

$$B_{+k_\parallel} = A_{-1}e^{-i(G-k_\parallel)x} + A_0 e^{ik_\parallel x} + A_1 e^{+i(G+k_\parallel)x}$$

And

$$B_{-k_\parallel} = A_{-1}e^{-i(G+k_\parallel)x} + A_0 e^{-ik_\parallel x} + A_1 e^{+i(G-k_\parallel)x}$$

Since these two symmetric modes are independent Bloch modes, they are orthogonal. Thus, in symmetric structures, there are at least two competing modes for lasing off the Γ point, as opposed to only one at the Γ point.

Furthermore, notice that the components A_{-1} and A_1 do not have the same wavevector - the component A_1 has $|k_x| = |G + k_\parallel|$ and the component A_{-1} has $|k_x| = |G - k_\parallel|$. Therefore, these two components cannot form a standing wave. Nevertheless, it is possible to achieve off-Γ point accidental BICs with zero group velocity, which may involve higher order plane waves, making them less favourable to establish lasing, compared to the Γ point Bloch mode.

4 PMMA effect on field confinement and Q-factor

4.1 PMMA thickness requirement

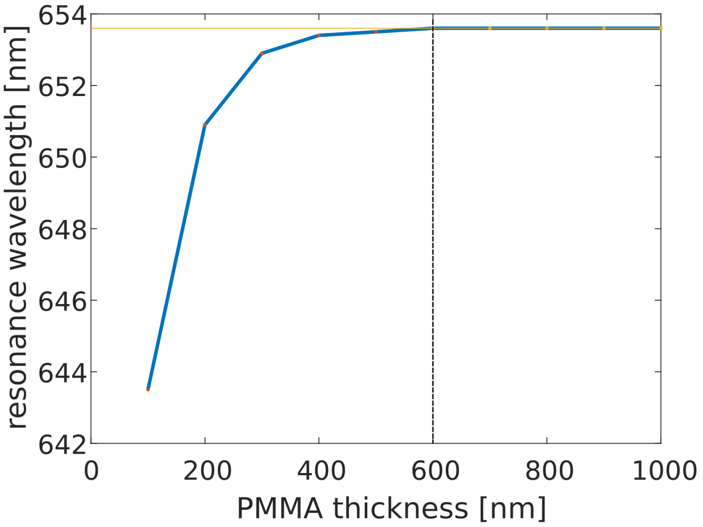

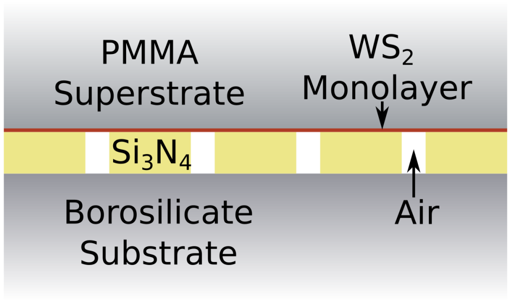

Figure S6. PMMA thickness simulation.(left)S^4-simulation of PMMA thickness-dependent resonance wavelength; (right) schematic drawing of the laser device. S^4-simulations showing negligible resonance wavelength changes above a PMMA thickness of $\approx$ 400 nm.

4.2 Effect of PMMA layer on field confinement in the monolayer

Field confinement calculations in COMSOL

To investigate the influence of the PMMA layer on the field confinement within the nanohole array structure, we performed rigorous coupled-wave analysis (RCWA) using a commercially available software (Ansys Lumerical Solutions). We assumed an infinite rectangular array by applying periodic boundary conditions to the lateral directions of a single nanohole unit cell. The resonance frequencies for the TM GMR mode have been determined for the nanohole array metastructure, with and without the PMMA layer, and the cross-sectional electric field magnitude along the longitudinal direction in each case has been calculated at the maximum magnitude location within the unit cell (see Fig. S7).

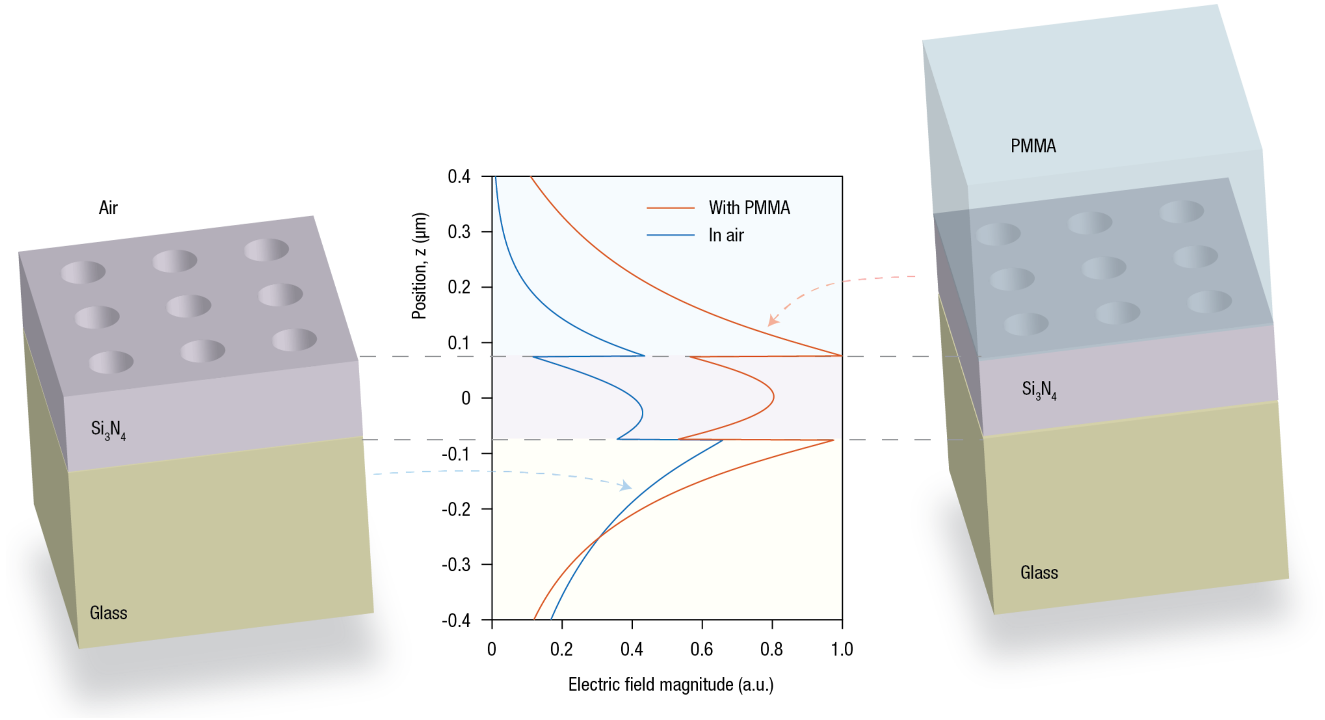

Figure S7. **Effect of PMMA layer on field confinement.** A 2-times stronger enhancement with PMMA at the Si_3N_4-PMMA interface, compared to the Si_3N_4-Air interface.

4.3 Effect of PMMA layer on Q-factors

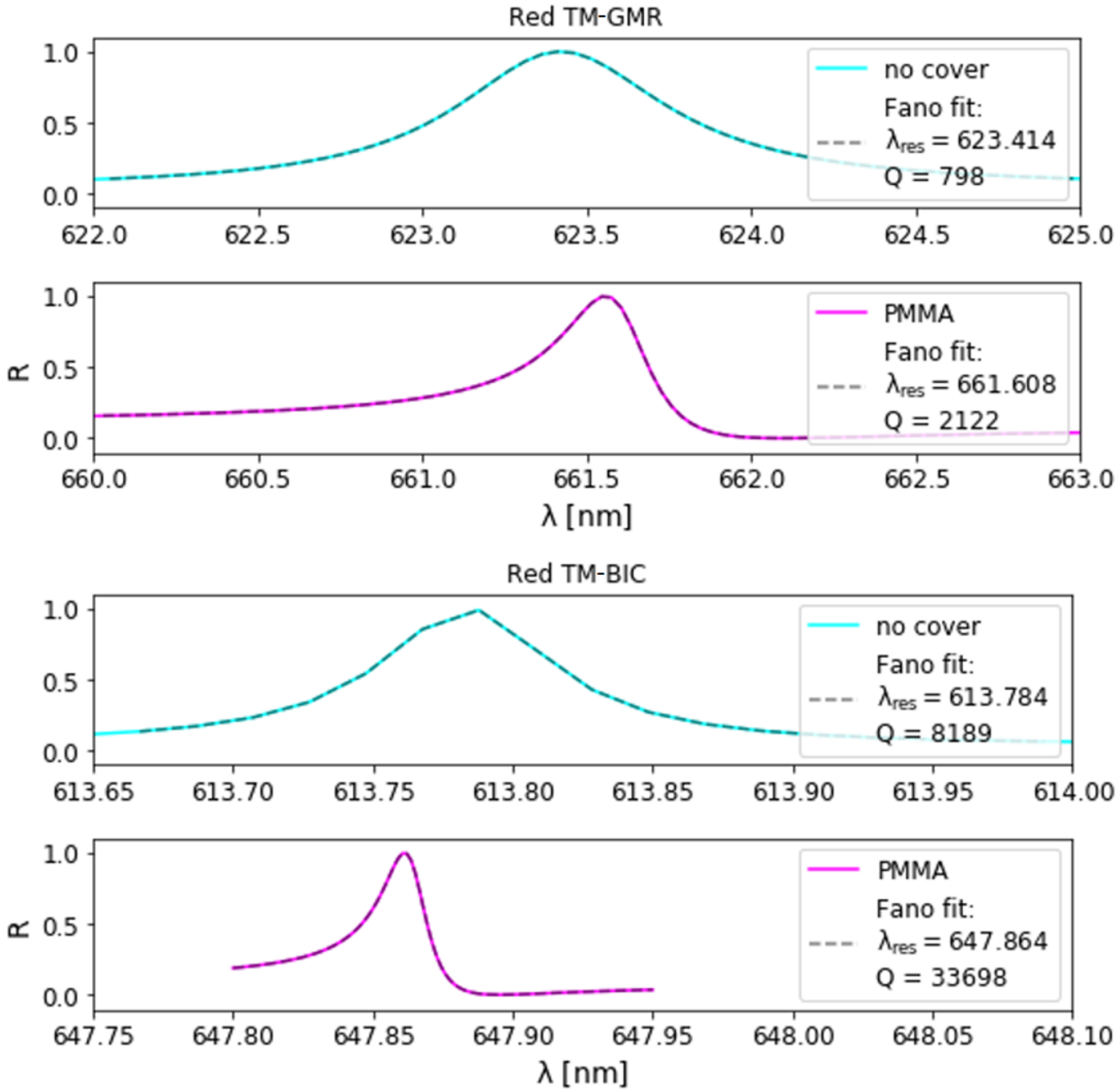

Figure S8. Effect on Q-factors in a zero-loss structure. Spectra with GMR and BIC peaks (0.5° incidence angle) in PMMA or air with extracted parameters from Fano fits. Without the PMMA layer, the Q factor of the TM-GMR and TM-BIC mode are 798 and 614, respectively. With the PMMA layer, the Q factor of the TM-GMR and TM-BIC mode are 2122 and 33698, respectively.

5 Q-factor effected by finite size and loss

5.1 Finite size of the device

All the simulations in this work were performed with infinite-sized structures with a periodic boundary condition, however, in reality the samples always had a finite size (typically 500 x 500 μm). To investigate the finite effect on the Q factor, we need to consider a finite structure. 3D Simulation of a nanohole metasurface with a large number of unit cells is computationally heavy. Instead, we simulated the Q-factor with different number of unit cells in a 1D grating structure without the PMMA layer to illustrate the finite size effect on Q, see Fig. S9. Once the size of the resonator exceeds 500 unit cells, the Q-factor is no longer limited by the finite size effect. Our laser cavity typically consists of more than 1100 unit cells.

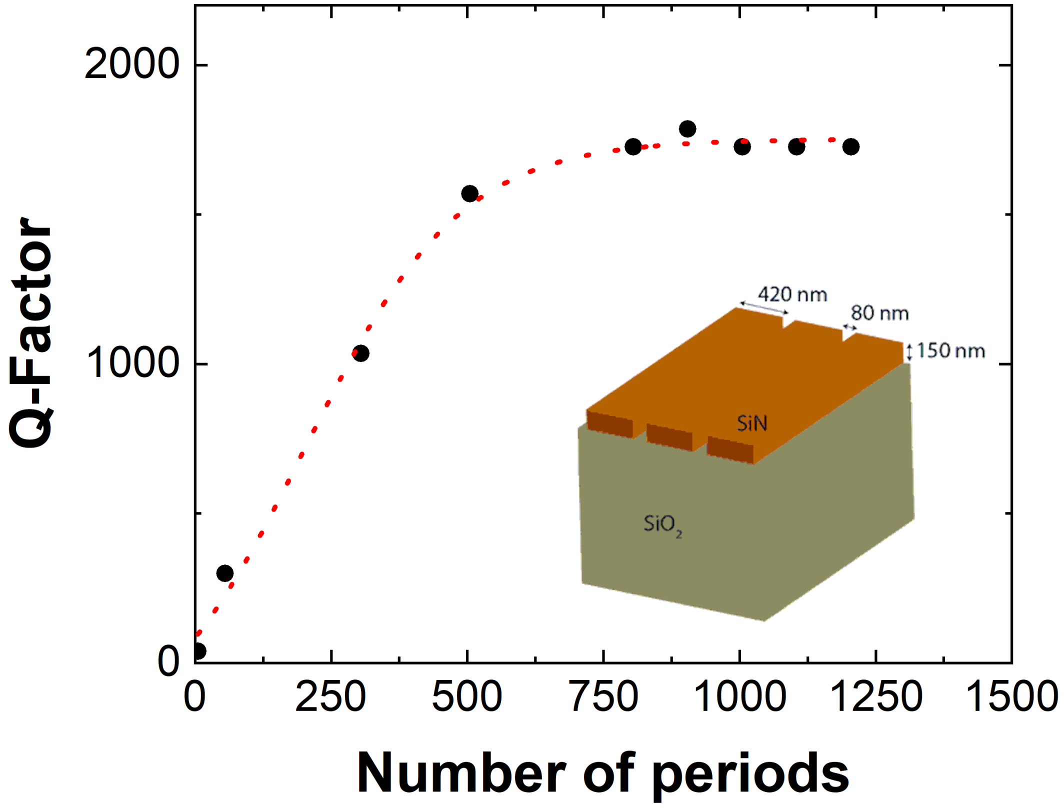

Figure S9. **Simulation of Q factors with increasing number of periods with a 1D grating structure. The dotted line is a guide to the eye.**

5.2 Loss limited Q-factors

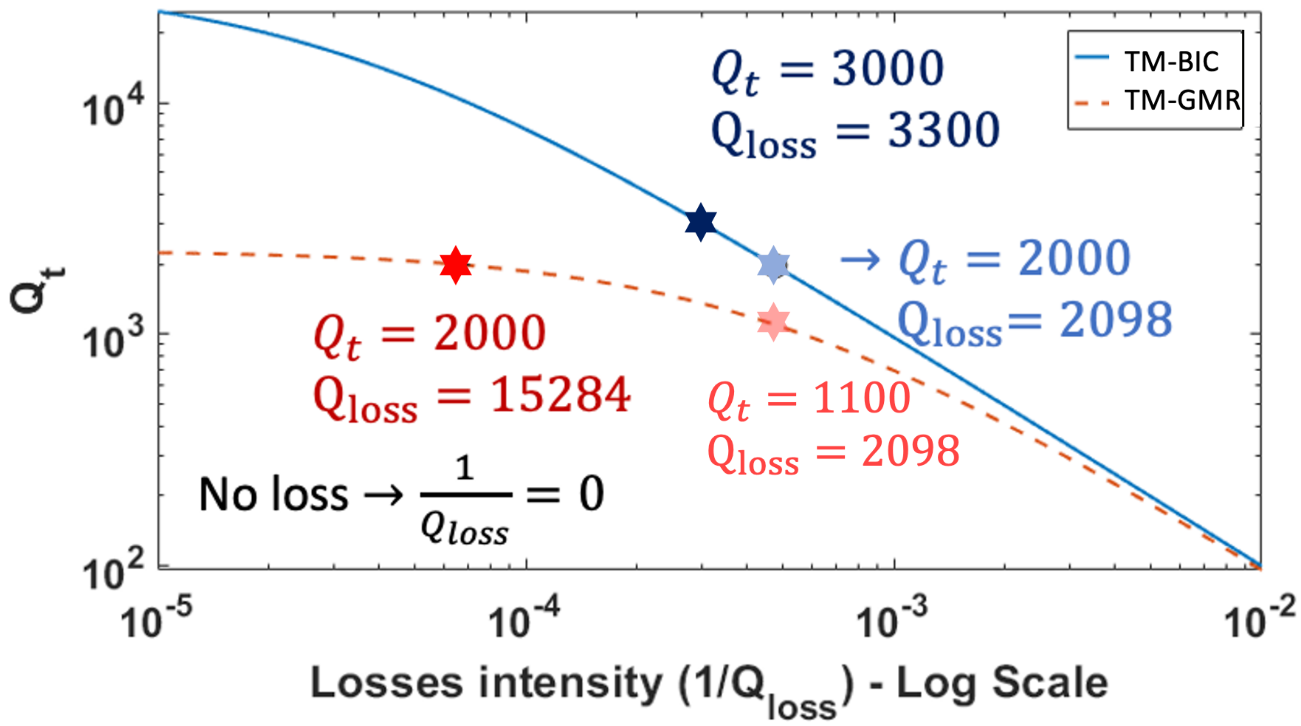

Figure S10. Simulation of Q-factors with a range of losses. The Q factor of the TM-BIC mode drops more dramatically than the TM mode. Here we consider that the scattering loss dominates in the laser devices.

6 Output power and quantum threshold condition

A general definition of the lasing threshold is at the point where stimulated emission overtakes spontaneous emission, suggested by Björk et al[2]. For a microcavity mode in an active gain medium, this will occur when the mean photon number in the mode is unity, known as the quantum threshold condition[3]. We can calculate the cavity lifetime as follows, assuming the best-case scenario when the laser has a spontaneous emission factor of 1 and the cavity is loss-less:

$$\tau = \frac{QT_{cycle}}{2\pi}, \quad \text{where} \quad T_{cycle} = \frac{\lambda}{c}$$

We take a wavelength λ of 650 nm, and a Q-factor of 3000, the photon lifetime in a cavity is then calculated to be approximately 6×10^{-13} s. This means that the cavity must emit a photon every 0.6 ps in order to meet the quantum threshold condition. Emitting a photon of 650 nm wavelength every 0.6 ps corresponds to an output power of approximately 500 nW. From a typical TM-GMR laser device, We measure an output power of >20 nW, knowing which is significantly underestimated because of the low NA (NA = 0.1) and the more than 50% loss of the power in the optical path in the setup.

7 Laser polarisation in the far field

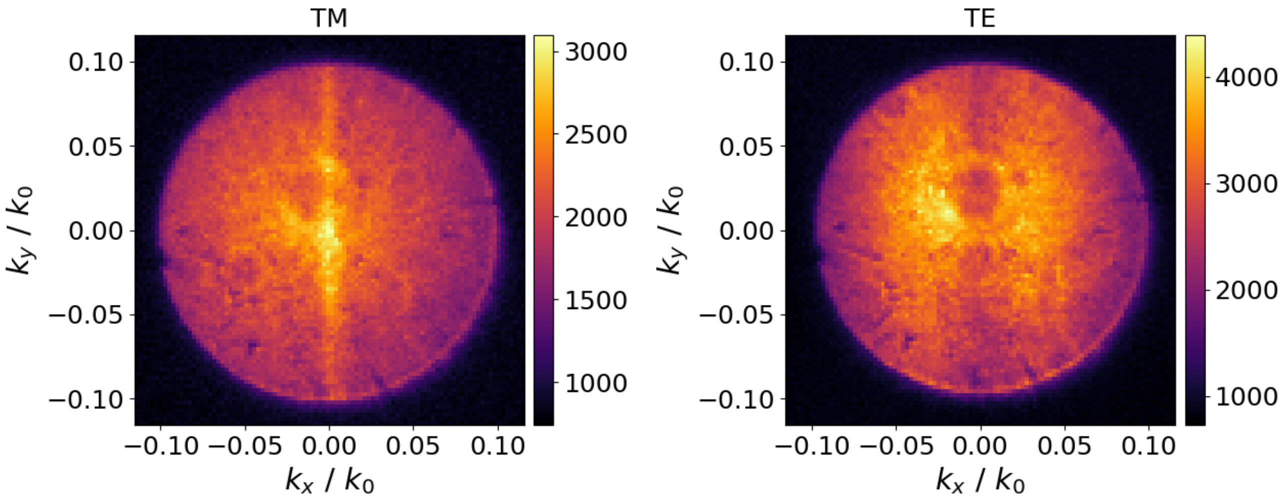

Figure S11. Laser emission with linear polariser in the imaging path. The laser line going through the Γ-point is TM-polarised and disappears for TE-polarisation.

8 Coherence and far field analysis

8.1 Far Field Beam Divergence Analysis

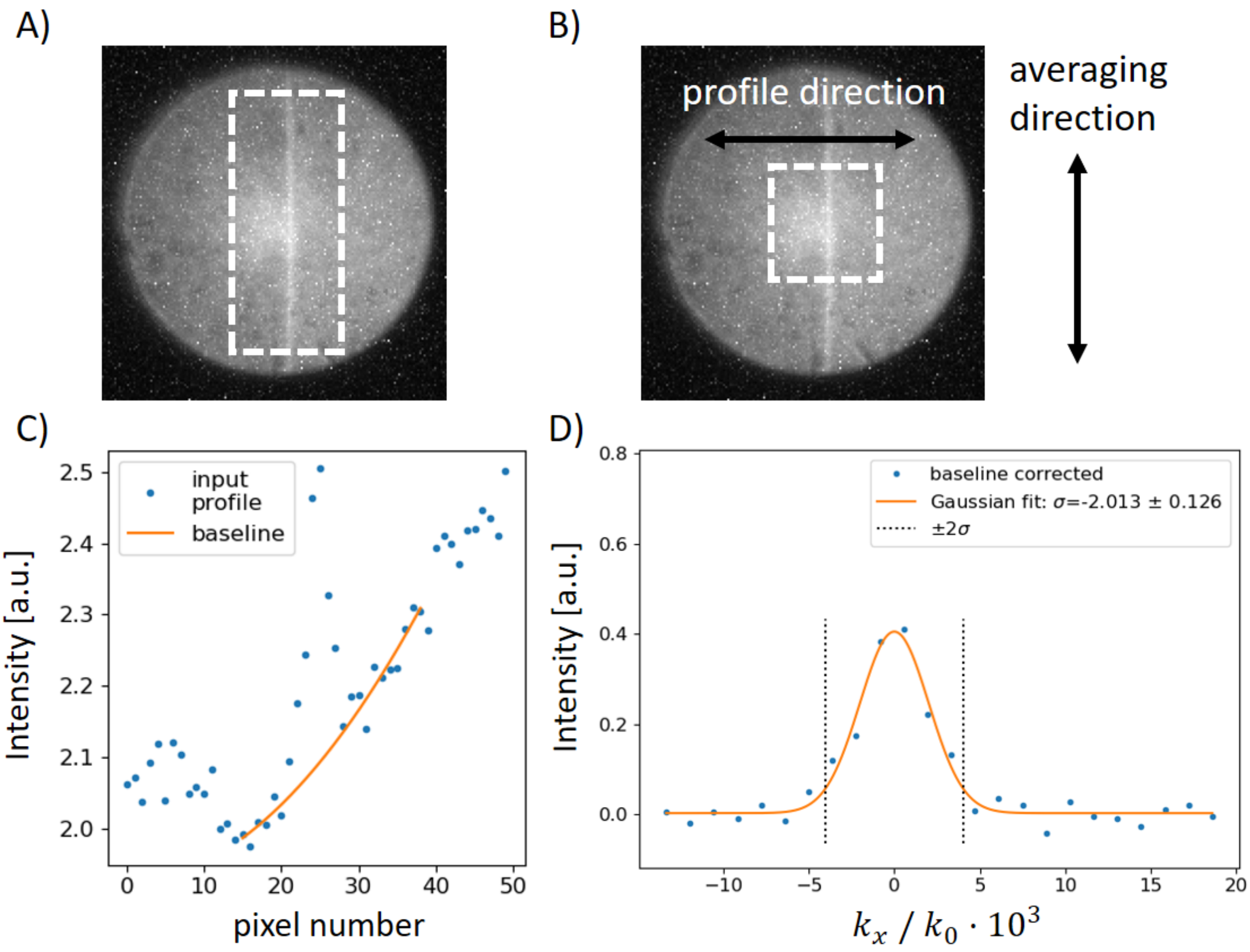

Figure S12. Extraction of divergence angles from back focal plane (BFP) images. BFP images of the laser emission with a big (A) and smaller (B) region of interest (ROI) to average. C) The extracted average beam profile with a baseline obtained by a quadratic fit of the data points next to the central peak. D) Background subtracted data with Gaussian fit. The x-axis transformed from pixel to angle using the radius of the NA circle. The divergence angle is taken as the angle at which the intensity drops by $1/e^2$ of the peak value, i.e. $\pm 2\sigma$.

sample number	$\sigma_{big\ ROI}$ [mrad]	$\sigma_{small\ ROI}$ [mrad]
1	2.6 ± 0.2	2.0 ± 0.1
2	2.6 ± 0.2	2.3 ± 0.2
3	3.3 ± 0.3	1.81 ± 0.08
4	5.1 ± 0.1	5.0 ± 0.1
5	1.79 ± 0.09	1.63 ± 0.09
6	1.7 ± 0.1	2.2 ± 0.2
7	1.4 ± 0.1	1.0 ± 0.2
mean	2.6 ± 0.5	2.3 ± 0.5

Table S1. Table of Gaussian σ extracted from BFP image profiles for seven different samples, averaging a small and a big ROI for each. On average, $\sigma_{big\ ROI}$ is 12 % higher than $\sigma_{small\ ROI}$, suggesting that the profile is either not perfectly linear and/or widens with distance from the centre. We obtain an average beam divergence beam divergence angle of $\theta = 2\sigma_{small\ ROI} = (4.6 \pm 1)$ mrad.

8.2 Additional double-slit experiment

In addition to the results obtained with a 200 μm slit separation, interferogram data was taken with a larger slit separation, i.e. 300 um, which corresponds to a distance of 45 μm on the sample. A fringe visibility of 0.12 was measured, see Figure S14.

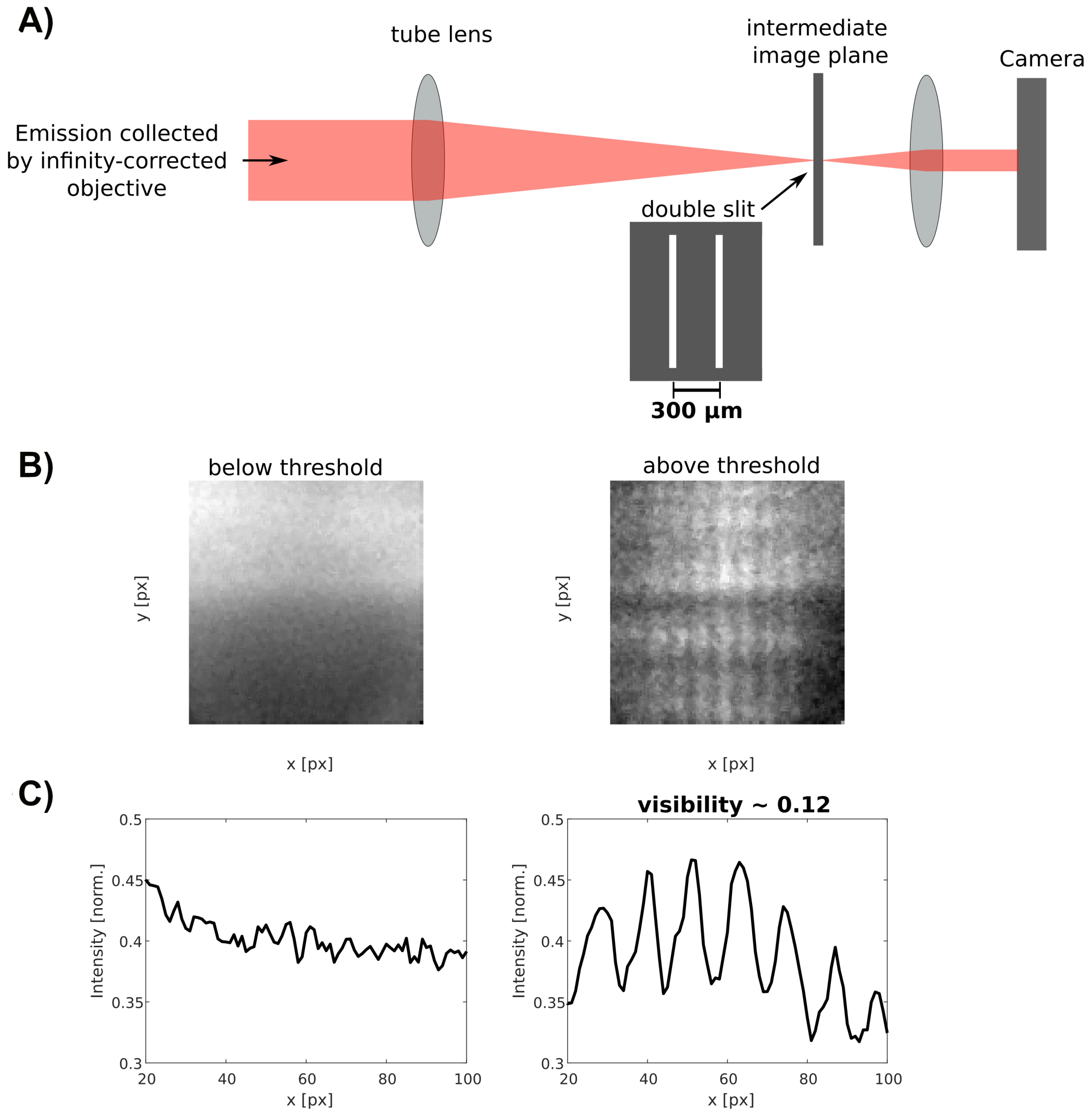

Figure S13. Characterisation of spatial coherence. A) Schematic of the double slit placed into an intermediate image plane; **B)** Interferograms recorded below and above threshold with a double slit distance of 300 μm, corresponding to a distance of 45 μm on the sample. **C)** Line profiles extracted from the interferograms.

8.3 Correlation between coherence length and far field measurement

Assuming a spatially coherent, rectangular lasing area of side lengths l_x and l_y, on the laser surface, the divergence angle can be calculated using the formula for the first minima in the diffraction pattern of a rectangular slit of the same dimensions that is illuminated by a plane wave. The corresponding angles are given by:

$$\theta_i = \pm \lambda / l_i, i \in \{x, y\}$$

From the angular spread presented in the far-field images in Fig. 4, we expect an upper bound of spatial coherence length of $l_x = 74\ \mu m$ (for TM-BIC with $\theta_x = 8.8$ mrad) and $l_x = 186\ \mu m$ (for TM-GMR with $\theta_x = 3.5$ mrad), which are higher than the measured coherence, i.e. the actual coherence of the devices could be even better than the measured values.

9 Wavelength tunability

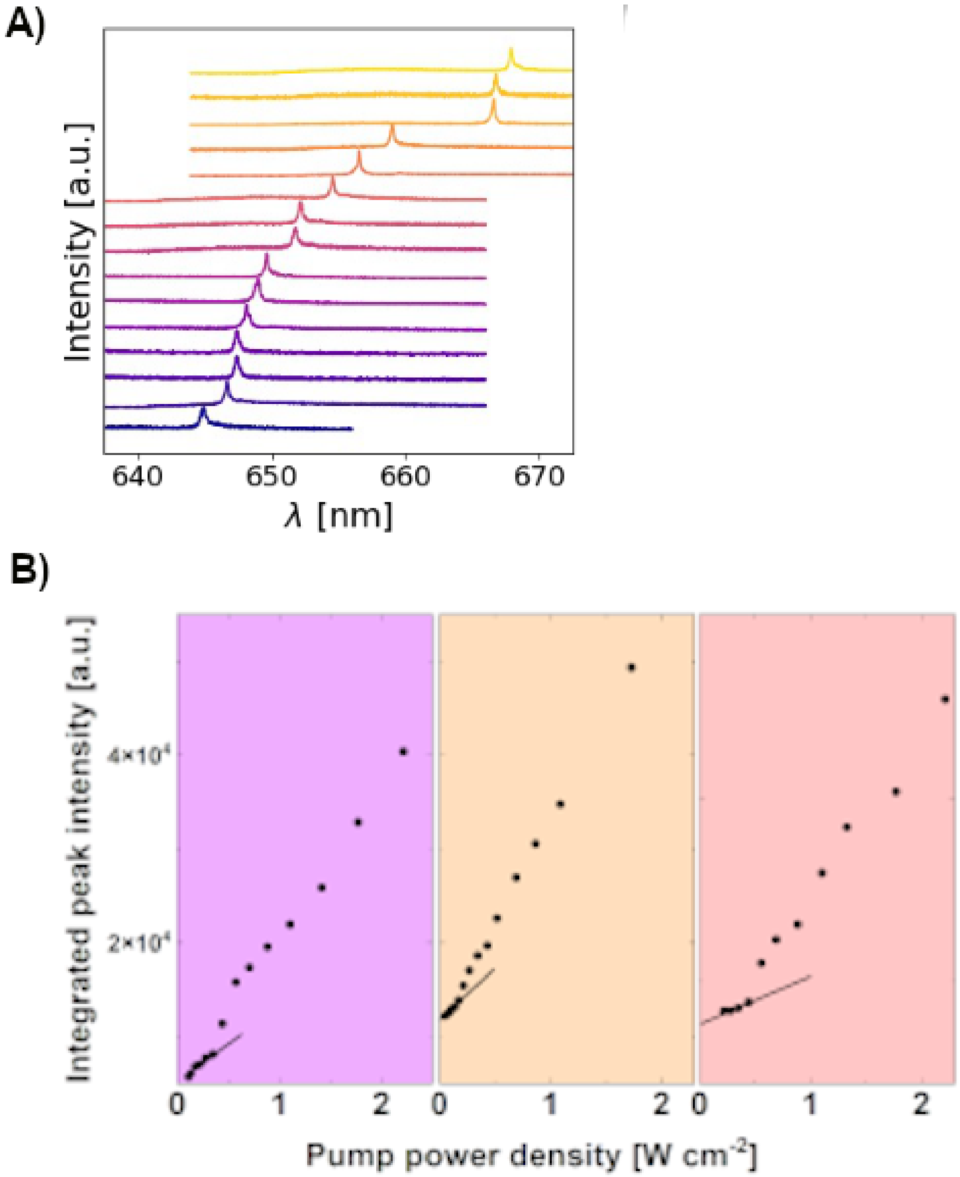

Figure S14. Additional lasing devices. A) Lasing spectra from all devices (above threshold). **B)** Light in-light out (L-L) curves from the three lasers in Fig. 3C. Spontaneous emission factor, β was estimated by fitting a rate equation calculation to the experimental data and from device to device, the β value varies in the range of 0.2 and 0.4.

References

1. Zheng, W. *et al.* Electrically pumped lasing from bound states in the continuum. *Res. Sq.* (2022).

2. Björk, G., Karlsson, A. & Yamamoto, Y. Definition of a laser threshold. *Phys. Rev. A* **50**, 1675 (1994).

3. Reeves, L., Wang, Y. & Krauss, T. F. 2d material microcavity light emitters: to lase or not to lase? *Adv. Opt. Mater.* **6**, 1800272 (2018).
